# Beneficial Propionibacteria within a Probiotic Emmental Cheese: Impact on Dextran Sodium Sulphate-Induced Colitis in Mice

**DOI:** 10.3390/microorganisms8030380

**Published:** 2020-03-07

**Authors:** Houem Rabah, Fillipe Luiz Rosa do Carmo, Rodrigo Dias de Oliveira Carvalho, Barbara Fernandes Cordeiro, Sara Heloisa da Silva, Emiliano Rosa Oliveira, Luisa Lemos, Denise Carmona Cara, Ana Maria Caetano Faria, Gilles Garric, Marielle Harel-Oger, Yves Le Loir, Vasco Azevedo, Guillaume Bouguen, Gwénaël Jan

**Affiliations:** 1 STLO, INRA, Agrocampus Ouest, 35 000 Rennes, France; houyemrabah1990@gmail.com (H.R.); gilles.garric@inra.fr (G.G.); marielle.harel-oger@inra.fr (M.H.-O.); yves.le-loir@inra.fr (Y.L.L.); 2 Pôle Agronomique Ouest, Régions Bretagne et Pays de la Loire, F-35 042 Rennes, France; 3 Departamento de Genética, Ecologia e evolução, Instituto de Ciências Biológicas, Universidade Federal de Minas Gerais (UFMG), Belo Horizonte, Minas Gerais CP 486 CEP 31270-901, Brazil; fillipelrc@gmail.com (F.L.R.d.C.); bafcordeiro@gmail.com (B.F.C.); saraheloisas@gmail.com (S.H.d.S.); emiliano.roliv@gmail.com (E.R.O.); luisalemossantos@gmail.com (L.L.); deniseccm@gmail.com (D.C.C.); anacaetanofaria@gmail.com (A.M.C.F.); vascoariston@gmail.com (V.A.); 4 I.M. Sechenov First Moscow State Medical University (Sechenov University), Moscow 119146, Russia; rodrigodoc2@gmail.com; 5 CHU Rennes, Univ Rennes, INSERM, CIC1414, Institut NUMECAN (Nutrition Metabolism and Cancer), F-35000 Rennes, France; Guillaume.BOUGUEN@chu-rennes.fr

**Keywords:** probiotic, colitis, cheese, inflammation, propionibacteria, Emmental, intestine, inflammatory bowel disease

## Abstract

Backgrounds and Aims. Inflammatory Bowel Diseases (IBD), including Ulcerative Colitis (UC), coincide with alterations in the gut microbiota. Consumption of immunomodulatory strains of probiotic bacteria may induce or prolong remission in UC patients. Fermented foods, including cheeses, constitute major vectors for bacteria consumption. New evidences revealed anti-inflammatory effects in selected strains of *Propionibacterium freudenreichii*. We thus hypothesized that consumption of a functional cheese, fermented by such a strain, may exert a positive effect on IBD. Methods. We investigated the impact of cheese fermented by *P. freudenreichii* on gut inflammation. We developed an experimental single-strain cheese solely fermented by a selected immunomodulatory strain of *P. freudenreichii*, CIRM-BIA 129. We moreover produced, in industrial conditions, an Emmental cheese using the same strain, in combination with *Lactobacillus delbrueckii* CNRZ327 and *Streptococcus thermophilus* LMD-9, as starters. Consumption of both cheeses was investigated with respect to prevention of Dextran Sodium Sulphate (DSS)-induced colitis in mice. Results. Consumption of the single-strain experimental cheese, or of the industrial Emmental, both fermented by *P. freudenreichii* CIRM-BIA 129, reduced severity of subsequent DSS-induced colitis, weight loss, disease activity index and histological score. Both treatments, in a preventive way, reduced small bowel Immunoglobulin A (IgA) secretion, restored occludin gene expression and prevented induction of Tumor Necrosis Factor α (TNFα), Interferon γ (IFNγ) and Interleukin-17 (IL-17). Conclusions. A combination of immunomodulatory strains of starter bacteria can be used to manufacture an anti-inflammatory cheese, as revealed in an animal model of colitis. This opens new perspectives for personalised nutrition in the context of IBD.

## 1. Introduction

Functional foods are defined as “ingredients that affect beneficially one or more target functions in the body, beyond adequate nutritional effects, in a way that is relevant to either an improved state of health and well-being and/or reduction of the risk of a disease” [1,2]. The dairy fermented foods, including cheeses, constitute a large part of our daily diet. Several investigations recently showed that specific bacteria strains of starter bacteria, typically employed in dairy fermented foods, can exert probiotic properties such as microbiota modulation, anti-cancerous and anti-inflammatory effects, in a strain-dependent manner. In this perspective, the development of functional dairy fermented foods by using probiotic starter bacteria, may constitute a promising manner to reduce the risk of diseases, which are related to lifestyle and diet. Some lactic acid bacteria and dairy propionibacteria strains were characterized for their immunomodulatory properties, specifically in Inflammatory Bowel Diseases (IBD) [3,4]. IBD, including Ulcerative Colitis (UC) and Crohn’s disease, are thought to result from a dysregulated innate and adaptive immune response towards the gut microbiome, in genetically susceptible host [5,6,7]. Ingested probiotic microorganisms may play a favourable role in the treatment of UC [8,9]. The consumption of specific food-grade microorganisms, selected for their immunomodulatory properties, alone or in combination with conventional drugs, was shown to induce and/or to enhance remission in UC patients [10].

New evidences [11,12] revealed the anti-inflammatory potential of selected strains of *Propionibacterium freudenreichii*, which is a cheese ripening starter routinely used, in association with lactic acid bacteria, in the production of Swiss-type cheeses such as Emmental cheese [13]. *P. freudenreichii* contributes to Emmental’s characteristic flavour and openings. It produces valuable metabolites with anti-inflammatory properties such as short chain fatty acids, 1.4-dihydroxy-2-naphthoic acid (DHNA), conjugated fatty acids, and surface proteins, which are produced in food matrices such as cheese [3]. Selected strains of *P. freudenreichii* were shown to induce the production of IL-10 in human peripheral blood mononuclear cells [12,14]. This in vivo immunomodulatory property correlates with the ability of these selected strains to protect from Trinitrobenzenesulfonic acid (TNBS)-induced colitis in mice [14]. This anti-inflammatory effect is mediated by specific surface proteins, found only at the surface of specific strains of *P. freudenreichii* [15,16]. A dairy food matrix was shown to protect such immunomodulatory surface proteins from digestive proteolysis [17,18]. Furthermore, consumption of experimental cheese fermented by *P. freudenreichii*, alone or with *Lactobacillus delbrueckii* subsp. *lactis*, protected mice from acute colitis induced by TNBS [19,20]. This led to limited induction of colitis markers such as serum IL-6, serum Amyloid A, colonic myeloperoxidase activity, as well as colonic mRNA expression level of *Il6, Tnfa, Il1b, Il10, Cox2* and *Hmox*. By contrast, colonic mRNA expression level of *Zo1, Pparg* and *Ifng*, repressed by TNBS, were restored by *P. freudenreichii* CIRM-BIA 129 consumption [14,19,20]. In accordance with our previous results, propionibacteria were recently reported to be enriched in the microbiota of infants as a result of breast-feeding, which attenuates the incidence of necrotizing enterocolitis. These authors isolated *Propionibacterium* UF1, closely related to *P. freudenreichii*, from healthy children and described it as a commensal *Propionibacterium* mitigating intestinal inflammation, via Th17 cell regulation and, regulating neonatal intestinal immunity [11,21].

Similar properties were reported for lactic acid bacteria, which may be used for Emmental cheese manufacturing [22]. Regarding all these data, we hypothesized that selection of specific lactic acid bacteria and dairy propionibacteria strains presenting anti-inflammatory properties could lead to a potentially probiotic Emmental favouring the treatment of IBD [19,20,23,24]. Therefore, the aim of this study was to evaluate the beneficial impact of an Emmental, made using three selected anti-inflammatory strains *P. freudenreichii* CIRM-BIA 129 [20], *Lactobacillus delbrueckii* subsp. *lactis* CNRZ327 [23], and *Streptococcus thermophilus* LMD-9 [25], in the context of Dextran Sodium Sulphate (DSS)-induced colitis in mice.

## 2. Materials and Methods

### 2.1. Bacterial Strains

*Lactobacillus delbrueckii* subsp. *lactis* CNRZ327, *Propionibacterium freudenreichii* CIRM-BIA 129 (equivalent to ITGP20 strain) and *Streptococcus thermophilus* LMD-9, were provided by the international microbiological resource centre CIRM-BIA (Centre International de Ressources Microbiennes, Bactéries d’Intérêt Alimentaire). Lactobacilli were cultured in MRS (de Man, Rogosa, Sharpe) medium (Difco^TM^ Lactobacilli MRS Broth, Difco Laboratories, Becton, Dickinson and Company, Sparks, MD, USA) as described [26]. Thermophilic streptococci in M-17 as described [27]. Dairy propionibacteria in YEL (Yeast Extract Lactate) as described [28]. Except for MRS (Difco Laboratories), all bacterial reagents were from Biokar Diagnostics, Beauvais, France.

### 2.2. Cheeses Manufacturing for Animal Studies

Two kinds of cheeses were made for animal studies, (1) a single-strain experimental cheese fermented by *P. freudenreichii* only and (2) an industrial Emmental cheese fermented by *Lactobacillus delbrueckii subsp. lactis*, *Streptococcus thermophilus* and *P. freudenreichii*.

(1) The experimental single-strain cheese, solely fermented by *P. freudenreichii* CIRM-BIA 129, was prepared as previously described [19,20]. Briefly, this probiotic strain was grown in a sterilised cow milk, supplemented with milk proteins, milk cream and casein peptone, to generate an experimental pre-cheese reaching 10^9^ colony forming units (CFU)/mL, pH 5.5). This was then subjected to coagulation (chy-max^®^Extra, Chr. Hansen, Hørsholm, Denmark), cutting, heating (10 min, 40 °C), moulding, pressing (2 h, 37 °C), drying and wrapping. All these steps were performed under laminar flow. The biochemical composition of the cheese, determined as described previously [29,30], was: dry matter 58 g/100g, lipids 28 g/100g, proteins 29 g/100g, carbohydrates 0 g/100g, and calcium 840 mg/100g. As a germ-free control, a sterile control cheese matrix was prepared in the same way as the single-strain cheese, but without starter bacteria addition. In that aim, the sterilised cow milk, supplemented with milk proteins, milk cream and casein peptone, was acidified using Glucono Delta Lactone, prior to the same cheese manufacturing procedure, as described previously [20]. Propionibacteria were enumerated on Yeast-Extract-Lactate-Agar (YELA). Coliforms, mesophilic flora, thermophilic flora, yeast, and molds, all below 10 CFU/g, were also enumerated respectively according to KF, NF ISO 4832, NF ISO 4833, NF V 08–059, and NF V 08–059 method (Table S1).

(2) The probiotic Emmental cheese was manufactured at an industrial scale (a 80 kg cheese wheel) by Entremont Alliance^©^ Company (Malestroit, France) using their production standard process. *Lactobacillus delbrueckii* subsp. *lactis* CNRZ327, *Streptococcus thermophilus* LMD-9 and *P. freudenreichii* CIRM-BIA 129, all 3 provided by CIRM-BIA, were used as starters. Lactobacilli were enumerated by CFU counting on MRS-agar at 42 °C under anaerobiosis [30], streptococci on M17-agar at 42 °C [30] and propionibacteria on lithium-glycerol-agar at 30 °C under anaerobiosis [31] as described previously. To check that the propionibacterial strain recovered after cheese making was identical to that used as a starter, a strain-specific PFGE analysis was applied to isolated colonies as described previously [31,32,33]. Briefly, chromosomal DNA samples were prepared according to Gautier et al. [32] and digested using *Xba* I. Electrophoresis was run at 14 °C on a 1% agarose gel on a Chef DR II system (Bio-rad, Richmond, UK) with the following parameters: initial time 2s, final time 20s, migration time migration 20h, voltage 6V.cm^−1^ = 200V. For thermophilic lactobacilli, parameters were: enzyme *Asc*I [34], initial time 1s, final time 10s, migration time 16h, Voltage: 6V.cm^−1^ = 200V. For thermophilic streptococci, parameters were: enzyme *Sma*I [35], initial time 2s, final time 20 s, migration time 24h, Voltage: 6V.cm^−1^ = 200V. Gels are presented in the Figures S1–S3. As a routine control at Entremont Alliance^©^ Company, absence of *E. coli*, of coagulase^+^ Staphylococci, of Listeria and of Salmonella was checked using, respectively, the following media: RAPID’E.coli (Sanofi Diagnostics Pasteur, NF (Normes Françaises) ISO (International Organization for Standardization) 16649-2 norm), Baird Parker + RPF (Rabbit Plasma Fibrinogen) (NF ISO 6888-2/A1 norm), RLM (Rapid’L Mono, NF ISO 11290-2) and XLD (Xylose-Lysine-Désoxycholate) + compass Salmonella (NF ISO 6579-1/2017 norm).

### 2.3. Animals, Feeding Procedure and Dextran Sulfate Sodium Induced Colitis

**Figure 1:**
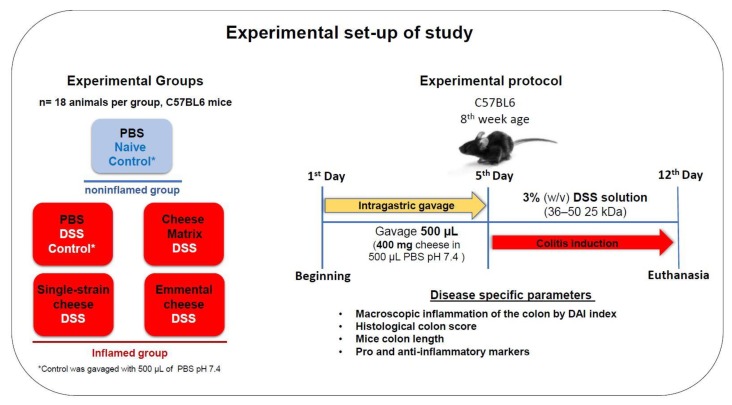
Experimental design of the evaluation of anti-inflammatory effects of a preventive intervention implementing *P. freudenreichii*-fermented cheeses, in the context of DSS-colitis. C57BL6 mice were divided into 5 groups, receiving different pre-treatments for 5 days, prior to induction of colitis. Colitis was then induced using 3% DSS in drinking water for 7 days prior to euthanasia. Different disease parameters were monitored to study the severity of colitis.

The experimental set-up of the animal study is depicted in Figure 1. Female C57BL6 mice (8 weeks old,) were obtained from Federal University of Minas Gerais (UFMG–Belo Horizonte, Brazil). The study was approved (11/03/2019) by the Brazilian Ethics Committee on Animal Use (CEUA-UFMG, Brazil, protocol 364/2018). They were randomly divided into groups of six and housed in a controlled environment (with a temperature of 25 °C, a 12 h/12 h light/dark cycle and ad libitum access to food and water). For the in vivo experiment, animals were divided into 5 groups of 18 C57BL6 mice. One control group received no DSS (naïve control) and four other groups received DSS. Mice were gavaged daily with 400 mg (per day per animal) of cheese prepared as described above, or with PBS (Phosphate-Buffered Saline). Cheeses were suspended in PBS buffer pH 7.4. Firstly, 400 mg of cheese were resuspended in 500 μL PBS and homogenised with the aid of the IKA T 10 Basic Ultra Turrax homogeniser probe (IKA^®^-Werke GmbH & Co. KG, Staufen, Germany) for 2–3 min. Mice were fed by intragastric gavage for seven consecutive days: 500 µl of PBS buffer, or 400 mg of the germ-free dairy matrix, or 400 mg of single-strain cheese or 400 mg of Emmental cheese. This amount of cheese was set to provide 10^9^ CFU of *P. freudenreichii*, for the single-strain cheese and the Emmental cheese, as described previously [19,20]. The maximal volume given daily by gavage was set according to the good practice guide to the administration of substances [36]. Then, DSS-colitis was induced by adding 3% dextran sulfate sodium (DSS) (36–50 kDa, CAT 260110, LOT Q5756 MP Biomedicals, Illkirch-Graffenstadenn, France), to the drinking water for 7 days. Among each group of 18 mice, all mice were analysed for weight loss, colon length and DAI (disease activity index) as indicated below. Among each group of 18 mice, 6 mice were randomly selected for histological analysis using the Swiss-roll technic, 6 other mice were randomly selected for RT-PCR (Reverse Transcription Polymerase Chain Reaction) analysis of gene expression, and 6 other mice were randomly selected for ELISA quantification of cytokines.

As a negative control, for each preventive treatment (germ-free cheese matrix control, single-strain *P. freudenreichii* cheese or industrial Emmental cheese), one group constituted of 6 mice was gavaged accordingly and left without DSS prior to euthanasia.

### 2.4. Assessment of DSS-Induced Colitis

The severity of colitis was assessed for 7 days before animal sacrifice. Weight loss, stool consistency and blood content were assessed. The DAI (disease activity index) score was calculated based on these markers, as previously described [37]. For each parameter, a score was given: absent (0), mild (1), moderate (2) and severe (3). The disease activity index corresponds to the sum of different scores.

### 2.5. Histology

Histomorphological analyses were conducted as follows. Briefly, the distal portion of the mice colon was collected after the euthanasia and gently washed with PBS. Colon tissue samples were immersed in formaldehyde solution (4% *v*/*v*) for tissue fixation (Sigma-Aldrich, St. Louis, MO, USA). The material was then embedded in paraffin, and a 4μm section of each sample was placed on a glass slide and stained with Hematoxylin-Eosin (Sigma-Aldrich). Slide images from each experimental group were captured (using a 20x objective) on a Spot Insight Color digital camera attached to the Olympus BX-41 Microscope using SPOT^®^ version 3.4 capture software. The score was determined according to McCafferty et al. [38], as the following features: extent of destruction of normal mucosal architecture (0: normal; 1: mild; 2: moderate; and 3: extensive damage), presence and degree of cellular infiltration (0: normal; 1: mild; 2: moderate; and 3: transmural infiltration), extent of muscle thickening (0: normal; 1: mild; 2: moderate; and 3: extensive thickening), presence or absence of crypt abscesses (0: absent; 1: present) and the presence or absence of goblet cell depletion (0: absent; 1: present). Mice were scored blindly by an expert pathologist.

### 2.6. Quantification of Secretory IgA in the Small Bowel Content

After mice sacrifice, the small bowel was collected. The content was washed using 10 mL of PBS. The intestinal contents were then vortexed and centrifuged (850× *g*, 30 min, 4 °C) as previously described [39]. The pellet was discarded while IgAs were quantified in the supernatant. Measurement of the levels of secretory IgA (sIgA) were determined by Enzyme-Linked Immunosorbent Assay (ELISA) in small bowel intestinal fluids. Microtiter plates Nunc-Immuno Plates, MaxiSorpTM (Nunc, Roskilde, Denmark) were coated with goat antibodies directed against mouse IgA, diluted 1:2000 in coating buffer antibodies (Southern Biotechnology, Birmingham, AL, USA) for 18 h at 4 °C. The plates were washed with saline (NaCl 0.9%) added with Tween 20 (0.05%) (Vetec, Rio de Janeiro, Brazil) and blocked with 200 µL PBS-casein (0.05%) for 1 h at room temperature. Intestinal fluid samples were diluted in PBS-casein (0.25%) and then added to the plate. After incubation for 1 h at room temperature, the wells were washed and biotin-conjugated anti-mouse IgA antibody (Southern Biotechnology Associates, Inc., Birmingham, AL, USA) diluted in PBS-casein (0.25%) (1: 10,000) and incubated for 1 h at 37 °C. Then, peroxidase-conjugated streptavidin (1:10,000) was added (Southern Biotechnology Associates, Birmingham, AL, USA). After 1 h of incubation, 100 µl of orthophenylenediamine (OPD) (Sigma-Aldrich) and H_2_O_2_ (0.04%) were added to each well. Plates were kept away from light until the coloration developed. The reaction was stopped by addition of 2N H_2_SO_4_. Reading was performed on a Model 450 Microplate Reader (Bio-Rad, Philadelphia, PA, USA), at 492 nm absorbance. The results were measured in concentration of sIgA (µg) per ml of intestinal fluid, according to the standard curve.

### 2.7. Gene Expression Analysis in the Distal Colon

The distal colon was cut into 1 cm fragments which were collected and stored in RNAlater at -80 °C until RNA extraction according to [40]. Total RNA was isolated using RNeasy mini kit (Qiagen, Hilden, Germany) according to the manufacturer’s instructions. Residual DNA was digested by adding RNas-free DNase I (Thermofisher Scientific, Bordeaux, France). Samples were then treated with Turbo DNA free Kit^®^ (Thermofisher Scientific). cDNA for each sample was produced with high capacity cDNA Reverse Transcription kit (Thermofisher Scientific). Quantitative PCR (Polymerase Chain Reaction) was performed using iTaq universal SYBR green supermix (Thermofisher Scientific) and by using gene specific primers for colonic cells (Table S3). Actin and GAPDH genes were used as housekeeping genes. Amplification reactions were performed on an ABI PRISM 7900HT Sequence detection system (Thermofisher Scientific). The amplification cycle consisted of the following steps: 95 °C for 30 s, and 40 cycles of 95 °C for 15 s and 60 °C for 30 s. The results of gene expression of the control group (with no treatment) were used as calibration data. Expression levels are represented as fold changes (2^−∆∆Ct^), using the means and standard deviation of target genes.

### 2.8. Tissues Preparation and Cytokines Quantification by ELISA

Colon fragments (100 mg for each mouse) were homogenized in 1 mL of PBS buffer containing 0.05% tween-20 (Vetec, Rio de Janeiro, Brazil), 0.1 mM phenylmethylsulfonyl fluorid (MP Biomedicals, Solon, Ohio, USA), 0.1 mM benzethonium chloride (Sigma-Aldrich), 10 mM EDTA (ethylenediaminetetraacetic acid) (Synth, Brazil) and 20 KIU aprotinin A (Sigma-Aldrich). Tissues mixtures were centrifuged (3.000× *g*, 10 min) and supernatants were collected for ELISA immunoassays using DuoSet ELISA kit (R&D Systems, Minneapolis, MN, USA). Plates (Nunc^®^, Sigma-Aldrich) were coated with purified monoclonal antibodies anti IL-10, IL-1β, IL-12p70, IL-17, IFN-γ, TGF-β, TNF-α and IL-6, overnight at 4 °C. Plates (Nunc-Immuno Plates, MaxiSorp) were washed by TBS (Tris-buffered saline) and supernatant from homogenized colon tissues were added. Plates then were incubated overnight at 4 °C. After plates washing, biotinylated monoclonal antibodies against different cytokines were added to coated plates and incubated for 2 h at room temperature. The revelation was performed by adding 100 µl/well of a citrate buffer containing Orthophenyldiamine (Sigma-Aldrich) (1 mg/mL) and 0.04% (*v*/*v*) H_2_O_2_. Then, 2N H_2_SO_4_ solution was added to stop the reaction. The absorbance was measured at 492 nm using an ELISA reader (Bio-Rad, Philadelphia, PA, USA).

### 2.9. Statistical Analysis

The protective effect of the different cheeses in the DSS-induced colitis preventions data was analysed using two-way (weight monitoring) and one-way (all other biomarkers monitoring) ANOVA followed by Tukey multiple comparisons test. Statistical significance was set at *p* < 0.05. Statistical analyses were performed in GraphPad Prism version 7.00 for Windows (GraphPad Software, San Diego, CA, USA.). All data were expressed as mean values and standard deviation (SD).

## 3. Results

### 3.1. Both Experimental and Industrial Emmental Cheeses Contain P. freudenreichii CIRM-BIA 129

Microbiological analyses showed that *P. freudenreichii* CIRM-BIA 129 grew up to 1.10^10^ and up to 4.10^9^ CFU/g, in the experimental single-strain cheese and in the industrial Emmental cheese, respectively (Table S1). The experimental single-strain cheese contained *P. freudenreichii* CIRM-BIA 129 as the only bacterium. In Emmental, dairy propionibacteria constituted the main bacterial population, above 10^9^ CFU/g, while lactobacilli and streptococci were much lower, close to 10^6^ CFU/g. In Emmental, the identity of dairy propionibacteria was checked by Pulsed-field gel electrophoresis (PFGE) (Table S2 and Figure S1). *P. freudenreichii* CIRM-BIA 129 was the only propionibacterial strain present in the Emmental cheese. When looking at thermophilic lactobacilli, different strains were identified, but *L. delbrueckii* CNRZ327, used as starter, was predominant (60%). For *S. thermophilus*, four different strains were found and the starter strain LMD-9 was not the predominant one (Table S2 and Figures S2 and S3). These are non-starter lactic acid bacteria.

### 3.2. Emmental Cheese Mitigates DSS-Induced Colitis in Mice

In these experiments, we assessed the preventive effect of *P. freudenreichii*, consumed either alone in a single-strain experimental cheese, or together with *S. thermophilus* and *L. delbrueckii* in an industrial Emmental cheese, in the context of DSS-induced colitis in mice. Mice received 400 mg of cheese per day, which corresponds to a dose close to 10^9^ live propionibacteria per day. Figure 1 illustrates the experimental set-up of the experiment. General biomarkers of DSS-induced colitis severity were mitigated, as described below, in the context of colitis. As expected, the different cheeses (germ-free cheese matrix, single-strain cheese or Emmental cheese) failed to modify these biomarkers in healthy mice in the absence of DSS (data not shown).

### 3.3. Disease Activity Index, Body Weight Loss, Colon Length and Histological Score in DSS-Colitis Mice

**Figure 2:**
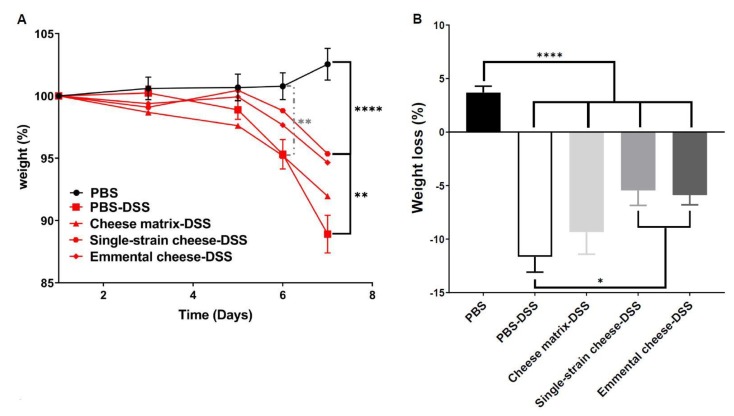
Impact of cheese matrix, single-strain cheese and Emmental cheese on colitis-induced body weight loss. (**A**) Time-course of mice body weight monitoring, and differences across groups. (**B**) Body weight loss observed at the 7th day of DSS colitis induction, and differences across groups. Groups were as follows. PBS: healthy group gavaged using PBS buffer as a sham. PBS-DSS: DSS-treated-colitis control group gavaged using PBS buffer as a sham. Cheese matrix-DSS: DSS-treated group gavaged using a germ-free dairy matrix. Single-strain cheese-DSS: DSS-treated group gavaged using an experimental single-strain cheese containing *P. freudenreichii* CIRM-BIA 129 as a sole bacterium. Emmental-DSS: DSS-treated group gavaged using an industrial Emmental cheese produced using *P. freudenreichii* CIRM-BIA 129 as a ripening starter. The data represent the mean ± SD of 18 mice per group. Multiple comparisons were performed, * *p* < 0.05, ** *p* < 0.01, *** *p* < 0.001 **** *p* < 0.0001.

**Figure 3:**
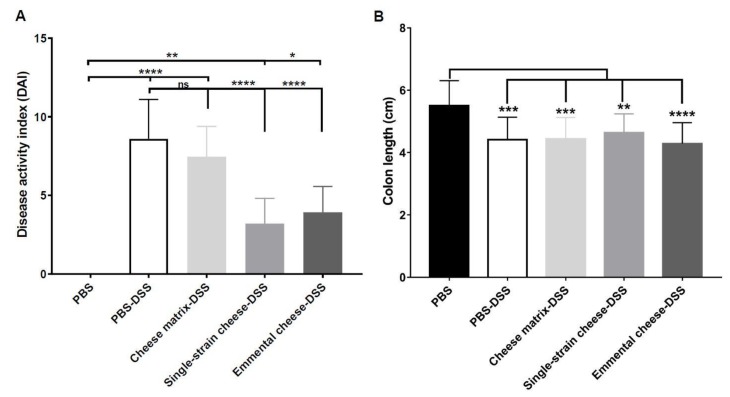
Impact of cheese matrix, single-strain cheese and Emmental cheese on the severity of DSS-induced colitis. Seven days after colitis induction, disease activity index (DAI) (**A**) and Colon length (**B**) were determined. Groups were as follows. PBS: healthy group gavaged using PBS buffer as a sham. PBS-DSS: DSS-treated-colitis control group gavaged using PBS buffer as a sham. Cheese matrix-DSS: DSS-treated group gavaged using a germ-free dairy matrix. Single-strain cheese-DSS: DSS-treated group gavaged using an experimental single-strain cheese containing *P. freudenreichii* CIRM-BIA 129 as a sole bacterium. Emmental-DSS: DSS-treated group gavaged using an industrial Emmental cheese produced using *P. freudenreichii* CIRM-BIA 129 as a ripening starter. The data represent the mean ± SD of 18 mice per group. Multiple comparisons were performed, * *p* < 0.05, ** *p* < 0.01, *** *p* < 0.001 **** *p* < 0.0001.

**Figure 4:**
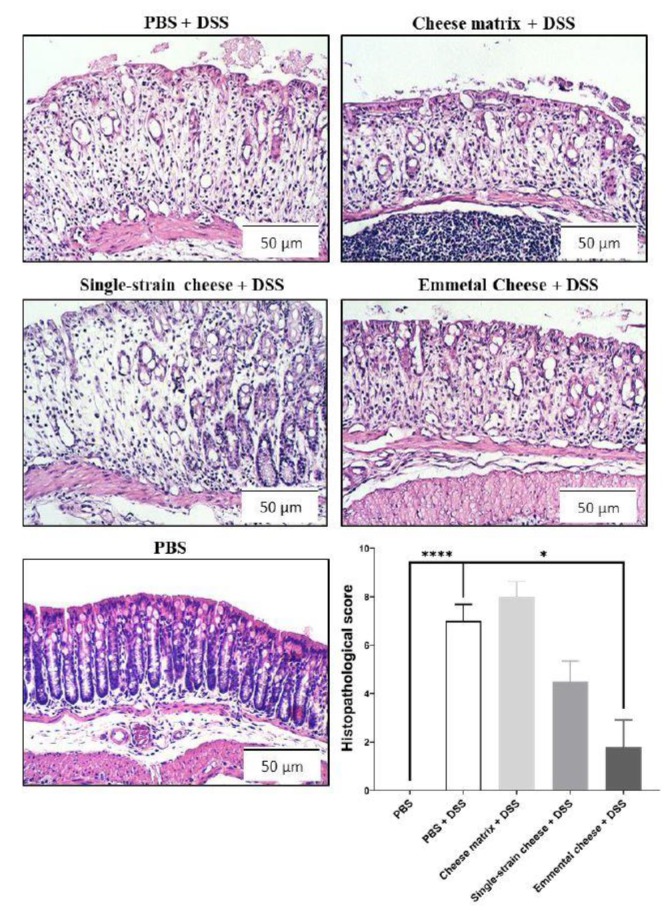
Impact of cheese matrix, single-strain cheese and Emmental cheese on DSS-induced histopathological damages. Representative images of mice colon mucosa sections, stained with haematoxylin, are shown. Image acquisition phase was done with a 20× magnification objective. Scale bar = 50 μm. Histopathological scores were determined. Groups were as follows. PBS: healthy group gavaged using PBS buffer as a sham. PBS-DSS: DSS-treated-colitis control group gavaged using PBS buffer as a sham. Cheese matrix-DSS: DSS-treated group gavaged using a germ-free dairy matrix. Single-strain cheese-DSS: DSS-treated group gavaged using an experimental single-strain cheese containing *P. freudenreichii* CIRM-BIA 129 as a sole bacterium. Emmental-DSS: DSS-treated group gavaged using an industrial Emmental cheese produced using *P. freudenreichii* CIRM-BIA 129 as a ripening starter. The data represent the mean ± SD of 6 mice per group. Multiple comparisons were performed, * *p* < 0.05, ** *p* < 0.01, *** *p* < 0.001 **** *p* < 0.0001.

DSS-induced colitis caused significant body weight loss in all mice groups, compared to the healthy group (PBS) at days 6 and 7(Figure 2A). However, consumption of the single-strain cheese and of the Emmental cheese attenuated the body weight loss, compared to the PBS-DSS and to the cheese matrix-DSS groups (Figure 2A). Indeed, at day 7, the weight of mice consuming both cheeses were significantly different from that of control colitis mice, while consumption of the placebo cheese matrix failed to limit weight loss. More precisely, Emmental cheese consumption significantly limited weight loss: −5.882% ± 3.275 (*p* < 0.05), compared to control group DSS: −11.65% ± 5.368 (Figure 2B). By contrast, cheese matrix failed to limit weight loss (Figure 2B). As expected, DSS-induced colitis increased the disease activity index (DAI), which takes into account the body weight loss, the severity of diarrhoea, and the presence of blood in faeces (Figure 3A). The cheese matrix did not reduce the DAI, whereas consumption of the single-strain cheese or of the Emmental cheese significantly reduced the DAI, compared to the PBS-DSS group (Figure 3A). DSS-induced colitis caused colon shortening in all mice groups (Figure 3B). The intake of the cheese matrix, the single-strain cheese or the Emmental cheese did not prevent the colon shortening (Figure 3B). Regarding histological analysis of the mice colon, a variation of the histopathological score, depending on the treatment, was observed. DSS exposure drastically affected the mucosal architecture with ulcerations and extent of muscle thickening of the colon, as well as inflammatory cell infiltration, oedema and goblet cell depletion. Histopathological score was null in control conditions, while it was strongly increased following DSS treatment (*p* < 0.0001) (Figure 4). Consumption of Emmental cheese, prior to colitis induction, significantly reduced (*p* < 0.05) this score (Figure 4), as evidenced by limited destruction of the mucosal architecture and limited degree of cellular infiltration, compared to PBS-DSS control group (Figure 4). Altogether, these results show that the Emmental cheese reduced the severity of DSS-induced colitis.

### 3.4. Mice Intestinal IgA Secretory Production

**Figure 5:**
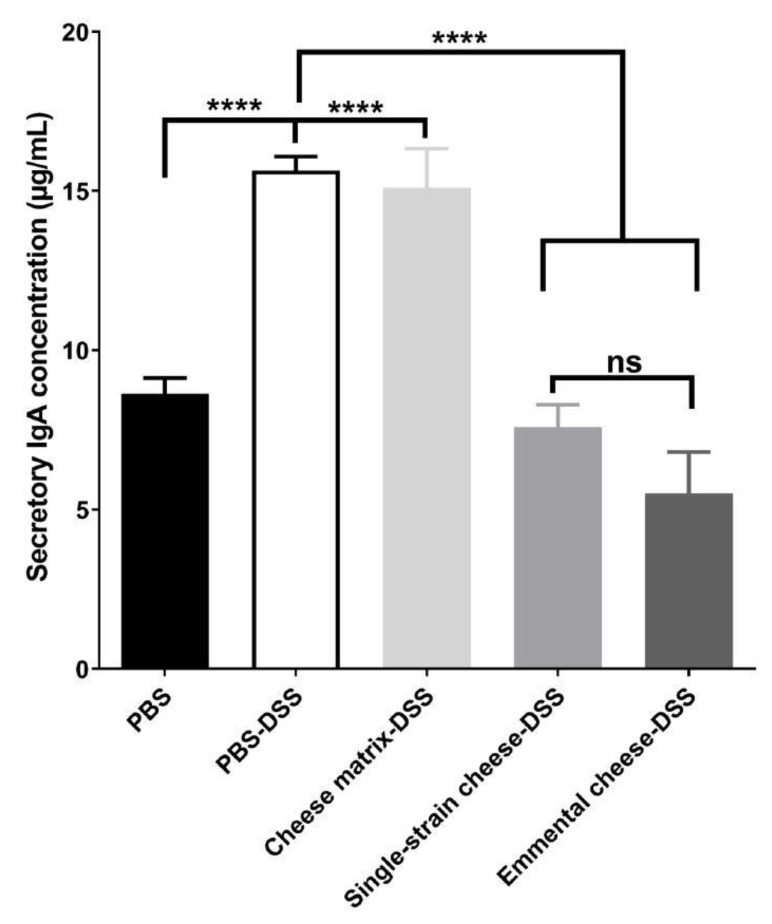
Impact of cheese matrix, single-strain cheese and Emmental cheese intake on small bowel IgA secretion. Secretory IgA concentration in the small bowel content was determined by ELISA quantification. The data represent the mean ± SD of 18 mice per group. Groups were as follows. PBS: healthy group gavaged using PBS buffer as a sham. PBS-DSS: DSS-treated-colitis control group gavaged using PBS buffer as a sham. Cheese matrix-DSS: DSS-treated group gavaged using a germ-free dairy matrix. Single-strain cheese-DSS: DSS-treated group gavaged using an experimental single-strain cheese containing *P. freudenreichii* CIRM-BIA 129 as a sole bacterium. Emmental-DSS: DSS-treated group gavaged using an industrial Emmental cheese produced using *P. freudenreichii* CIRM-BIA 129 as a ripening starter. Multiple comparisons were performed, * *p* < 0.05, ** *p* < 0.01, *** *p* < 0.001 **** *p* < 0.0001.

Concentration of secretory IgA in the small intestine of all mice groups was quantified using ELISA. The DSS-induced colitis increased significantly the IgA secretion, from 8.63 ± 1.11 to 15.63 ± 1.02 µg/mL (Figure 5 and Supplemental Table S4). Consumption of control cheese matrix did not attenuate IgA secretion, whereas consumption of single-strain cheese or of Emmental cheese reduced it significantly (7.59 ± 1.71 and 5.51 ± 3.15), compared to the PBS-DSS mice group (Figure 5). There was no significant difference, in terms of IgA secretion, between the single-strain cheese and the Emmental cheese (Figure 5).

### 3.5. Mice Colonic Oxidative Stress and Epithelial Barrier

**Figure 6:**
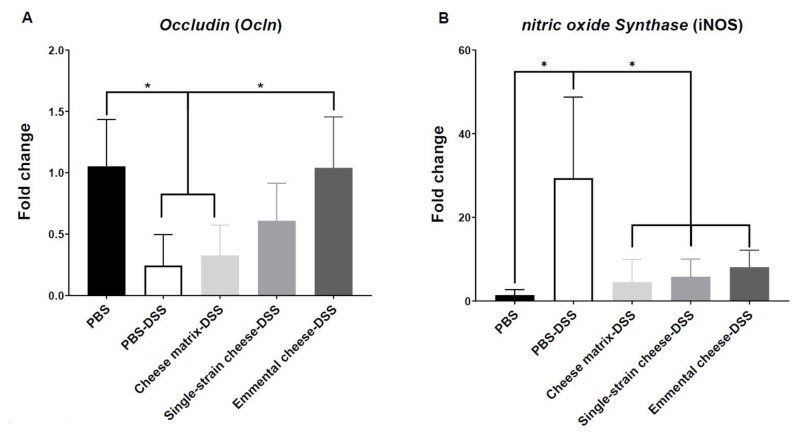
Impact of cheese matrix, single-strain cheese and Emmental cheese on colonic expression of markers of cell barrier and oxidative stress. Colonic mRNA expression levels of (**A**) Ocln and (**B**) iNOS genes were analysed. The data represent the mean ± SD of 6 mice per group. Groups were as follows. PBS: healthy group gavaged using PBS buffer as a sham. PBS-DSS: DSS-treated-colitis control group gavaged using PBS buffer as a sham. Cheese matrix-DSS: DSS-treated group gavaged using a germ-free dairy matrix. Single-strain cheese-DSS: DSS-treated group gavaged using an experimental single-strain cheese containing *P. freudenreichii* CIRM-BIA 129 as a sole bacterium. Emmental-DSS: DSS-treated group gavaged using an industrial Emmental cheese produced using *P. freudenreichii* CIRM-BIA 129 as a ripening starter. Multiple comparisons were performed, * *p* < 0.05, ** *p* < 0.01, *** *p* < 0.001 **** *p* < 0.0001.

Consumption of a pure culture of *P. freudenreichii* was previously shown to modulate colic expression of markers of inflammation, of oxidative stress, and of gut epithelial barrier integrity, in TNBS-colitis mice [19,20]. We sought here such modulation, as a result of *P. freudenreichii*-containing cheese, in DSS-colitis mice. The gene expression of different markers of the colonic oxidative stress and epithelial barrier integrity was assessed by quantitative RT-PCR. No significant change was observed regarding expression of genes involved in epithelial barrier integrity, as *claudind1*, *mucin 2*, *zonula occludens1* and *zonula occludens2* genes (data not shown). However, DSS-induced colitis triggered a significant decrease of *occludin* gene expression, which was prevented by consumption of Emmental cheese, yet not of the cheese matrix (Figure 6A). DSS-induced colitis triggered an oxidative stress in colonic cells as indicated by the induction of nitrite oxide synthase (*iNOS*) gene expression (Figure 6B). All the dairy products tested here, including the cheese matrix, the single-strain experimental cheese, and the Emmental cheese, prevented this *iNOS* induction.

### 3.6. Pro-Inflammatory and Anti-Inflammatory Gene Expression in Mice Colon

**Figure 7:**
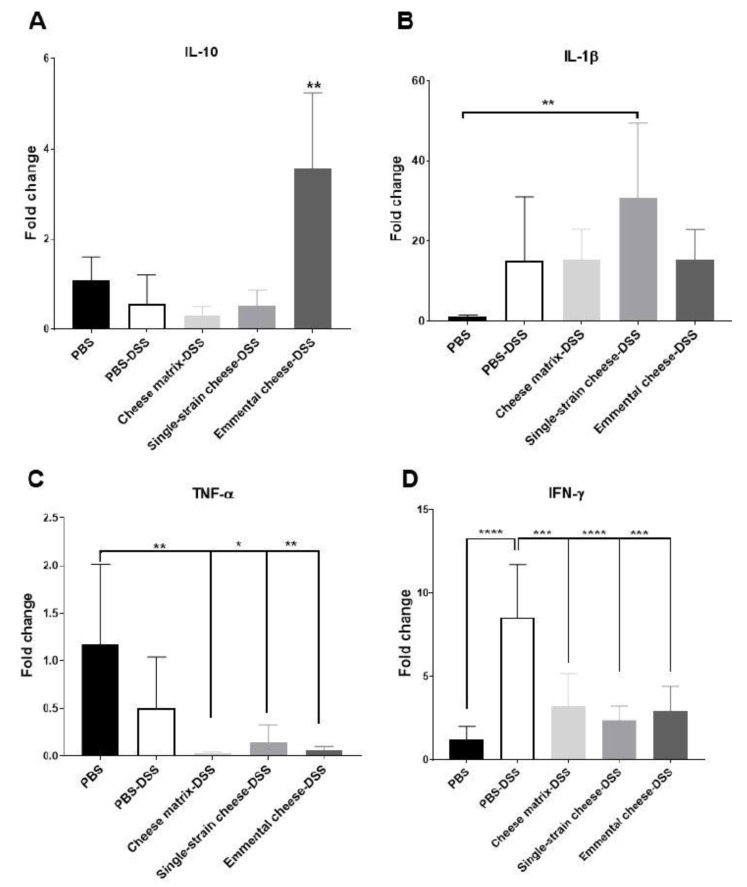
Impact of cheese matrix, single-strain cheese and Emmental cheese on colonic expression of cytokines genes during DSS-induced colitis. Colonic mRNA expression levels of (**A**) IL-10, (**B**) IL-1β, (**C**) TNFα and (**D**) IFNγ were determined. Data represent the mean ± SD of 6 mice per group. Groups were as follows. PBS: healthy group gavaged using PBS buffer as a sham. PBS-DSS: DSS-treated-colitis control group gavaged using PBS buffer as a sham. Cheese matrix-DSS: DSS-treated group gavaged using a germ-free dairy matrix. Single-strain cheese-DSS: DSS-treated group gavaged using an experimental single-strain cheese containing *P. freudenreichii* CIRM-BIA 129 as a sole bacterium. Emmental-DSS: DSS-treated group gavaged using an industrial Emmental cheese produced using *P. freudenreichii* CIRM-BIA 129 as a ripening starter. Multiple comparisons were performed, * *p* < 0.05, ** *p* < 0.01, *** *p* < 0.001 **** *p* < 0.0001.

Consumption of *P. freudenreichii* was also reported to modulate colic expression of cytokines in mice. We sought such modulation, as a result of *P. freudenreichii*-containing cheese, in DSS-colitis mice. The expression of pro-inflammatory and anti-inflammatory cytokines genes expression in the colonic cells was assessed in all mice groups. No significant modification of expression of *Tgfβ1* or of *IL21* was observed (data not shown). DSS-induced colitis did not modify *IL10* expression (Figure 7. A). Similarly, consumption of the cheese matrix and of the single-strain cheese did not change *IL10* and *Tgfβ1* expression (data not shown). However, consumption of Emmental cheese enhanced *IL10* expression in the DSS-induced colitis group, compared to all mice groups (Figure 7A). DSS-induced colitis did not induce significant change in *IL1β* and *Tnfα* expression in colonic cells, compared to the healthy group (Figure 7B,C). However, the single-strain cheese intake enhanced *IL1β* expression during DSS colitis, compared to the healthy group (Figure 7B). The cheese matrix, the single-strain cheese and the Emmental cheese decreased significantly *Tnfα* expression, compared to the healthy group (Figure 7C). In addition, these three dairy products all attenuated the increase of *Ifnγ* expression triggered by DSS (Figure 7D).

### 3.7. Pro-Inflammatory and Anti-Inflammatory Cytokines Concentration in Mice Colon

**Figure 8:**
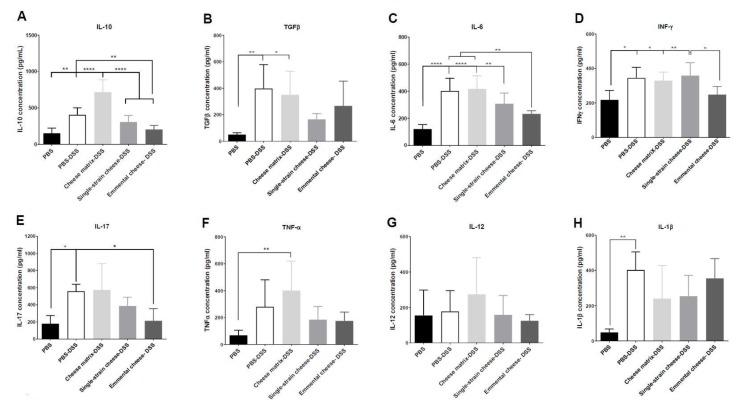
Impact of cheese matrix, single-strain cheese and Emmental cheese on colonic secretion of cytokines during DSS-induced colitis. Cytokines concentration of (**A**) IL-10, (**B**) TGFβ, (**C**) IL-6, (**D**) IFNγ, (**E**) IL-17, (**F**) TNFα, (**G**) IL-12 and (**H**) IL-1β were quantified by ELISA. Data represent the mean ± SD of 6 mice per group. Groups were as follows. PBS: healthy group gavaged using PBS buffer as a sham. PBS-DSS: DSS-treated-colitis control group gavaged using PBS buffer as a sham. Cheese matrix-DSS: DSS-treated group gavaged using a germ-free dairy matrix. Single-strain cheese-DSS: DSS-treated group gavaged using an experimental single-strain cheese containing *P. freudenreichii* CIRM-BIA 129 as a sole bacterium. Emmental-DSS: DSS-treated group gavaged using an industrial Emmental cheese produced using *P. freudenreichii* CIRM-BIA 129 as a ripening starter. Multiple comparisons were performed, * *p* < 0.05, ** *p* < 0.01, *** *p* < 0.001 **** *p* < 0.0001.

The concentration of pro-inflammatory and anti-inflammatory cytokines in the colonic tissues was assessed in all mice groups by ELISA quantification (Figure 8.). DSS-induced colitis increased IL-10 secretion, compared to the healthy group (Figure 8A). The cheese matrix intake further enhanced secretion of IL-10 during DSS-induced colitis, compared to the healthy group (Figure 8A). However, fermented cheeses consumption reduced IL-10 induction (Figure 8A). DSS-induced colitis increased TGFβ secretion in the PBS-DSS and the cheese matrix-DSS groups (Figure 8B). The Emmental cheese and the single-strain cheese ingestion did not influence significantly TGFβ secretion, compared to the DSS-PBS and the healthy groups (Figure 8B). Concerning IL-6, its concentration was increased during colitis, while this increase was prevented by Emmental cheese consumption (Figure 8C). Similarly, only Emmental cheese attenuated IFNγ secretion induced by the DSS-induced colitis (Figure 8D). DSS-induced colitis increased IL-17 secretion, compared to the healthy group, which is exacerbated by the cheese matrix intake (Figure 8E). Only Emmental cheese was able (*p* < 0.05) to decrease IL-17 secretion, compared to the PBS-DSS group (Figure 8E). DSS-induced colitis induced a significant increase of TNFα only in the cheese matrix group (Figure 8F). DSS-induced colitis did not induce significant change in IL-12 secretion, compared to the healthy group (Figure 8G). Cheese intake did not either alter significantly IL-12 secretion, compared to healthy group (Figure 8G). Finally, DSS-induced colitis increased IL-1β secretion compared to the healthy group, and this was not attenuated by the Emmental cheese intake (Figure 8H).

## 4. Discussion

Previous studies indicated the protective role of the consumption of selected strains of *Propionibacterium freudenreichii*, in the context of TNBS-induced colitis in mice [14,19,20]. In this study, we investigated the protective effect of the consumption of a “probiotic” Emmental cheese, in the context of DSS-induced colitis. This Emmental cheese was manufactured at an industrial scale in a local cheese maker’s plant and starter bacteria were selected for their immunomodulatory properties: *P. freudenreichii* CIRM-BIA 129 [20], *L. delbrueckii* subsp. *lactis* CNRZ327 [23] and *S. thermophilus* LMD9 [25]. At the end of ripening, propionibacteria constituted the main flora, above 10^9^ CFU.g^−1^, which is typical of Emmental cheese [41,42], and the strain *P. freudenreichii* CIRM-BIA 129 was the only one detected, as shown by PFGE analysis. Thermophilic streptococci and lactobacilli were much lower, close to 10^6^ CFU.g^−1^, which is also typical, as these bacteria grow up to 10^8^ to 10^9^ CFU.g^−1^, during curd fermentation, and then experience massive cell death during Emmental ripening [41,42]. Although *L. delbrueckii subsp. lactis* CNRZ327 was the predominant lactobacillus, they contained the strains added as starters, as well as other strains of thermophilic streptococci and lactobacilli. Indeed, non-starter lactic acid bacteria are usually found in Emmental [41,42,43].

In this study, Emmental probiotic cheese was compared to a previously described model of experimental single-strain cheese [19], fermented only by *P. freudenreichii* CIRM-BIA 129, and to a germ-free cheese placebo matrix, in a DSS-induced colitis model. No mortality was observed. DSS consumption caused an abrupt weight loss in the control group (DSS). However, the pretreatment with the Emmental cheese prevented it. Both Emmental cheese and single-strain cheese attenuated colitis severity and reduced inflammation, as evidenced by the disease activity index. However, only Emmental cheese was able to decrease the histopathological score compared to control group (DSS). These results are consistent with previous studies dealing with propionibacteria [19,20,23,24]. *P. freudenreichii* CIRM-BIA 129 alone, or in combination with lactic acid bacteria, attenuated TNBS-induced colitis [12,14,19,20]. However, neither of the two fermented cheeses prevented the colon shortening triggered by DSS. Similarly, in a previous study, consumption of *P. freudenreichii* CIRM-BIA 129 in combination with lactic acid bacteria did not prevent colon shortening during TNBS-induced colitis [44].

DSS-induced colitis increased IgA secretion in the small bowel, while both single-strain and Emmental cheeses attenuated this increase. Increased IgA secretion is the expression of ileal barrier disturbance by the inflammatory response. Reduced inflammation, as a result of cheese consumption, thus led to attenuated IgA response, suggesting that DSS-induced colitis would affect not only the large intestine but also the small intestine [45,46]. Accordingly, *P. freudenreichii* CIRM-BIA 129 was shown to reduced IgA response in the context of 5-fluorouracyl-induced mucositis [47]. Indeed, disruption of the gut barrier integrity is a key step of inflammatory bowel diseases [48]. DSS-induced colitis induced a significant increase of nitric oxide synthase (iNOS) expression in the colon tissues and this increase was attenuated by the three tested dairy products. However, only Emmental cheese restored colonic expression of the *Ocln* gene encoding the transmembrane protein *occludin*, which contributes to intestinal barrier function [49].

Cytokines are major mediators of colitis pathogenesis [5]. We thus assessed expression and concentration of cytokines in the colonic tissues. Results of cytokine expression were partially consistent with cytokine secretion results. DSS-induced colitis increased the secretion of IL-10 and of TGFβ, which is consistent with the observed induction of these cytokines in UC patients [50]. Both single-strain and Emmental cheeses attenuated this increase. However, only Emmental cheese, consumed as a protective pre-treatment, increased IL-10 gene expression in the colonic tissue, compared to all other groups. IL-10 is an anti-inflammatory cytokine which inhibits the production of IL-1β, IL-6, and TNF-α. Its increase has a protective effect towards colitis, only if it is triggered before DSS-colitis induction [51]. *L. delbrueckii subsp. lactis* CNRZ327, as well *P. freudenreichii* CIRM-BIA 129, were previously shown to increase, in animal models, the subset of Treg FOXP3+ cells, which produce a high amount of IL-10 [18,23,47]. Similarly, TGFβ is an anti-inflammatory mediator which is highly produced by mononuclear cells of UC patients [50]. Both single-strain and Emmental cheeses attenuated inflammation, and thus TGFβ secretion, in the context of DSS-colitis in this study.

Colitis increased secretion of IFNγ and of IL-6 and tended to increase the secretion of TNFα. At the expression level, only IFNγ expression was induced by DSS, compared to the healthy group. Consumption of the germ-free cheese matrix did not modulate the expression of TNFα, but decreased that of IFNγ, compared to the PBS-DSS group. However, the cheese matrix did not attenuate the secretion of IFNγ, TNFα and IL-6 triggered by the DSS. Both single-strain and Emmental cheeses attenuated expression of IFNγ gene but only Emmental cheese decreased the IFNγ secretion, compared to the PBS-DSS group. Similarly, Emmental cheese consumption decreased the secretion of IL-6. IFNγ, IL-6 and TNFα are pro-inflammatory cytokines highly secreted in the gut mucosa of UC patients [50]. TNF-α is produced by antigen-presenting cells and by macrophages. It induces the secretion of IL-6 and IFNγ, the typical cytokine of Th1 cells subset. IL-6 and TGFβ secretion can induce Th17 cells, which are involved in IBD pathogenesis [52,53]. Indeed, IL-17, the Th17 cells cytokine, was increased during DSS-induced colitis and tended to be attenuated (*p* = 0.06) by the Emmental cheese administration only.

Taken together, these results show that consumption of the complex Emmental cheese and of the single-strain cheese triggered different mechanisms. Propionic acid bacteria in Emmental cheese are predominant, while lactic acid bacteria undergo massive lysis during cheese ripening [54,55,56]. Killed lactic acid bacteria were, however, shown to modulate chemically-induced colitis [57,58,59,60]. The anti-inflammatory properties of lactic acid bacteria are probably mediated by cell wall components of lysed or dead cells. The interactions between propionic acid and lactic acid bacteria are poorly characterized and may modulate their probiotic properties. As an example, on one hand, the proteolytic activity of lactic acid bacteria can affect propionic acid bacteria immunomodulatory surface proteome, as well as their ability to produce beneficial metabolites [13,61]. On the other hand, lactic acid bacteria proteolysis may cause liberation of bioactive peptides from caseins, which can participate to the anti-inflammatory property of an Emmental cheese [62,63]. Further studies are needed to decipher interactions, between propionic and lactic acid bacteria, and how they affect their probiotic attributes. These studies will provide screening criteria to choose the most effective lactic and propionic acid bacteria to develop anti-inflammatory functional foods.

As a conclusion, an Emmental cheese, produced in industrial conditions, using well-characterized immunomodulatory starter strains, was able to mitigate the severity of DSS-induced colitis in a mice model. This protective effect seems to result from a synergy between lactic acid and propionic acid bacteria. It opens new perspectives for clinical studies on patients suffering from ulcerative colitis. Further clinical studies, implementing *P. freudenreichii*, should also take into account its interaction with the human gut microbiota. Previous studies reported a bifidogenic effect for propionibacteria consumption [64,65]. The modulation of other potent symbionts, including *Akkermansia*, *Lactobacillus* and *Faecalibacterium* species, should also be evaluated. Conversely, modulation of potent pathobionts, with a pro-inflammatory potential, deserves attention. As an example, sulfate-reducing bacteria were reported to participate to inflammation in experimental colitis [66]. They contribute to homeostasis disruption during intestinal inflammation, by promoting intestinal damage through generation of hydrogen sulfide at high levels [67]. Indeed, colitis correlates with a modified sulfate-reducing bacteria community in mice, exhibiting enhanced production of hydrogen sulphide [68]. This last may in turn affect the gut microbiota and probiotic efficacy [69]. Indeed, some lactobacilli, such as *L. reuteri*, *L. pentosus* and *L. paracasei*, may be extremely sensitive to hydrogen sulphide [70]. Conversely, lactic acid bacteria in the small bowel may provide lactate, which serves as an electron donor, while sulfate serves as an electron acceptor, in the production of hydrogen sulphide, as a toxic product in the small-large intestinal axis [71]. Its inhibitory effect on probiotic bacteria, including lactobacilli and propionibacteria, should thus be taken into account. Complex interactions between probiotic fermented dairy products and the human gut microbiota, as well as its metabolites, will determine their beneficial role in the context of gut inflammation.

## Supplementary Materials

Supplementary materials can be found at https://www.mdpi.com/2076-2607/8/3/380/s1. **Figure S1**: PFGE analysis of Propionibacterium freudenreichii clones, **Figure S2:** PFGE analysis of thermophilic streptococci clones, **Figure S3:** PFGE analysis of themophilic lactobacilli clones, **Table S1:** Microbiological analysis of the Emmental cheese, the single-strain cheese and the cheese matrix, **Table S2:** PFGE analysis of different colonies isolated from petri dishes done for *P. freudenreichii*, *Lactobacillus* and *S. thermophilus* enumeration in the Emmental cheese, **Table S3:** Specific primer sequences in order to target murine genes analysed in the study, **Table S4:** Secretory IgA in the different experimental mice groups.

## Abbreviations

Cox	Cyclooxygenase
DAI	Desease Activity Index
DSS	Dextran Sodium Sulphate
Hmox	Heme oxigenase
IFN	Interferon
Ig	Immunoglobulin
IBD	Inflammatory Bowel Disease
Th	T helper
TNF	Tumor Necrosis Factor
UC	Ulcerative Colitis
Zo1	Zonula occludens 1
Pparg	Peroxisome proliferator-activated receptor gamma
