# Supplementary material for: Beneficial Propionibacteria within a Probiotic Emmental Cheese: Impact on Dextran Sodium Sulphate-Induced Colitis in Mice
Source: Microorganisms. 2020 Mar 7;8(3):380. doi: 10.3390/microorganisms8030380 (PMC7142753; doi:10.3390/microorganisms8030380)
Supplement: Supplementary file 1 [file microorganisms-08-00380-s001.zip › SupplementalDataRevised.docx]

**Supplemental data**

**Table S1**. Microbiological analysis of the Emmental cheese, the single-strain cheese and the cheese matrix. *P. freudenreichii* enumeration was performed in the Emmental cheese and the Single-strain cheese. *Lactobacillus sp* and *Streptococcus thermophilus* numeration was done only in the Emmental cheese. Different microbiological analysis were performed to numerate contaminating flora in the Emmental cheese in one hand, and in the single-strain cheese and the cheese matrix in the other hand.

|  | **Emmental cheese** | | **Single-Strain cheese** | | **Cheese matrix** | |
| --- | --- | --- | --- | --- | --- | --- |
| ***P. freudenreichii*** | CFU / g | 4.10^9^ | CFU / g | 1.10^10^ | - | - |
| **Thermophilic *Lactobacillus*** | CFU / g | 1.10^6^ | - |  | - | - |
| ***Streptococcus thermophilus*** | CFU / g | 4.10^6^ | - |  | - | - |
| ***E. Coli*** | CFU / g | <10 | - |  | - | - |
| ***Staphylococcus*** | CFU / g | <10 | - |  | - | - |
| **Listeria** | CFU /25g | Not detected | - |  | - | - |
| **Salmonella** | CFU /25g | Not detected | - |  | - | - |
| **Total coliform Bacteria** | - | - | CFU / g | Not detected | CFU / g | Not detected |
| **Total thermophilic Bacteria** | - | - | CFU / g | <10 | CFU / g | <10 |
| **Total mesophilic Bacteria** | - | - | CFU / g | <10 | CFU / g | <100 |
| **Yeasts and molds** | - | - | CFU / g | Not detected | CFU / g | Not detected |

**Table S2**. PFGE analysis of different colonies isolated from petri dishes done for *P. freudenreichii*, *Lactobacillus* and *S. thermophilus* enumeration in the Emmental cheese. The identity of strains were checked by analyzing 10 different colonies isolated.

|  | **Number of colonies collected** | **Different strains identified** | **Colony corresponding to the original strain** |
| --- | --- | --- | --- |
| ***P. freudenreichii*** | 10 | 1 | 10/10 |
| ***L. delbrueckii*** | 10 | 4 | 6/10 |
| ***S. thermophilus*** | 10 | 4 | 1/10 |

**Table S3**. Specific primer sequences in order to target murine genes analysed in the study.

| Primer | Sequence 5’-3’ | | Reference |
| --- | --- | --- | --- |
| Ocln | Forward | GGACCCTGACCACTATGAAACAGACTA | ^42^ |
|  | Reverse | TAGGTGGATATTCCCTGACCCAGTC |  |
| CLD1 | Forward | CTGGAAGATGATGAGGTGCAGAA |  |
|  | Reverse | CTAATGTCGCCAGACCTGAA |  |
| Muc2 | Forward | GCTGACGAGTGGTTGGTGAATG | ^43^ |
|  | Reverse | GATGAGGTGGCAGACAGGAGAC |  |
| ZO1 | Forward | AGCTCATAGTTCAACACAGCCTCCAG | ^42^ |
|  | Reverse | CTTCCACAGCTGAAGGACTCACAG |  |
| ZO2 | Forward | GGAGACCAGATTCTGAAGGTGAACACA |  |
|  | Reverse | CCTTTGGGGATTTCTAGCAGGTAGAGGAC |  |
| iNOS | Forward | CAGCTGGGCTGTACAAACCTT | ^44^ |
|  | Reverse | CATTGGAAGTGAAGCGTTTCG |  |
| Actb | Forward | AGAGGGAAATCGTGCGTGAC |  |
|  | Reverse | CAATAGTGATGACCTGGCCGT |  |
| Gapdh | Forward | TCACCACCATGGAGAAGGC |  |
|  | Reverse | GCTAAGCAGTTGGTGGTGCA |  |
| IL1beta | Forward | CAACCAACAAGTGATATTCTCCATG |  |
|  | Reverse | GATCCACACTCTCCAGCTGCA |  |
| IL6 | Forward | GAGGATACCACTCCCAACAGACC |  |
|  | Reverse | AAGTGCATCATCGTTGTTCATACA |  |
| IL10 | Forward | GGTTGCCAAGCCTTATCGGA |  |
|  | Reverse | ACCTGCTCCACTGCCTTGCT |  |
| IL12b | Forward | GGAAGCACGGCAGCAGAATA |  |
|  | Reverse | AACTTGAGGGAGAAGTAGGAATGG |  |
| IL17a | Forward | GCTCCAGAAGGCCCTCAGA |  |
|  | Reverse | AGCTTTCCCTCCGCATTGA |  |
| Infg | Forward | TCAAGTGGCATAGATGTGGAAGAA |  |
|  | Reverse | TGGCTCTGCAGGATTTTCATG |  |
| Tgfb1 | Forward | TGACGTCACTGGAGTTGTACGG |  |
|  | Reverse | GGTTCATGTCATGGATGGTGC |  |
| Tnfa | Forward | CATCTTCTCAAAATTCGAGTGACAA |  |
|  | Reverse | TGGGAGTAGACAAGGTACAACCC |  |

**Table S4**. Secretory IgA in the different experimental mice groups

|  | PBS | PBS + DSS | Matrix + DSS | Single Strain cheese + DSS | Emmental + DSS |
| --- | --- | --- | --- | --- | --- |
| means secretory IgA | 8,63 | 15,63 | 15,11 | 7,59 | 5,51 |
| SD secretory IgA | 1,11 | 1,02 | 2,98 | 1,71 | 3,15 |

**Figure S1** PFGE analysis of *Propionibacterium freudenreichii* clones.

**Figure S2** PFGE analysis of thermophilic streptococci clones.

**Figure S3** PFGE analysis of themophilic lactobacilli clones.
